# Pseudomonas aeruginosa Mobbing-Like Behavior against Acanthamoeba castellanii Bacterivore and Its Rapid Control by Quorum Sensing and Environmental Cues

**DOI:** 10.1128/Spectrum.00642-21

**Published:** 2021-12-01

**Authors:** Nimrod Shteindel, Yoram Gerchman

**Affiliations:** a Department of Environmental and Evolutionary Biology, Faculty of Natural Science, University of Haifa, Haifa, Israel; b Department of Biology, Oranim College, Kiryat Tivon, Israel; University of Maryland School of Pharmacy

**Keywords:** adhesion, biofilm, PQS, predation avoidance, quorum sensing

## Abstract

Mobbing, group attack of prey on predator, is a behavior seen in many animal species in which prey animals use numbers and coordination to counter individually superior predators. We studied attack behavior of Pseudomonas aeruginosa toward the bacterivore Acanthamoeba castellanii. This behavior consists of directed motility toward and specific adhesion to the predator cells, enacted in seconds and responding to both prey and predator population densities. Attack coordination relies on remote sensing of the predator and the use of the Pseudomonas quinolone signal (PQS), a P. aeruginosa species-specific quorum sensing molecule. Mutants unable to produce the PQS show unspecific adhesion and reduced survival, and a corresponding increase in predator population occurs as a result of predation. The addition of an external PQS restored some predator-specific adherence within seconds, suggesting a novel response mechanism to this quorum sensing (QS) signal. Fast behavioral response of P. aeruginosa to PQS is also supported by the rate of signal accumulation in the culture, reaching relevant concentrations within minutes, enabling bacteria response to self population density in these short timescales. These results portray a well-regulated group attack of the bacteria against their predator, reacting within seconds to environmental cues and species-specific signaling, which is analogous in many ways to animal mobbing behavior.

**IMPORTANCE** Pseudomonas aeruginosa was shown previously to attack amoebae and other predators by adhering to them and injecting them with virulent substances. In this work, we show that an active, coordinated group behavior is enacted by the bacteria to utilize these molecular components, responding to both predator and bacterial population density. In addition to their ecological significance, immediate behavioral changes observed in response to PQS suggest the existence of a fast QS signal cascade, which is different from canonical QS that relies on slow-to-respond gene regulation. Similar regulatory circuits may drive other bacterial adaptations and pathogenicity mechanisms and may have important clinical implications.

## INTRODUCTION

Predation by protozoa is a major selective force acting on bacterial populations, producing a variety of predation avoidance mechanisms (1, 2); assembling into bigger and harder to swallow multicellular structures (3), swimming away from a predator (4), producing antipredator toxins (5), interfering with predator phagocytosis and digestion processes, and modulating cell surfaces to evade predator recognition (6).

Many animal species that face a similar problem evolved corresponding predation avoidance strategies. One of these strategies is mobbing in which a group of prey organisms attack a predator, employing numbers and coordination to counter individual predator superiority (7). Mobbing requires a sufficient number of prey organisms (8) and synchronization of attack behavior (9). This behavior was reported in a variety of species, including insects (10), fish (11), mammals (12), and birds (13), and was showed to be facilitated by visual (14), vocal (15), and chemical communication (16).

Pseudomonas aeruginosa, a bacterial species common in many environments, was shown to attack and kill phagocytic predators using an impressive chemical arsenal. Reports include killing of paramecia (17), flagellates (18), surface-associated ciliates (19), and several types of amoebae (18, 20, 21). Some of these attacks are facilitated by virulent substances secreted by the bacteria to the extracellular environment, while others rely on substances injected directly into the predator cell (22). Direct injection enables bacteria to produce a higher concentration of the virulent substances in the confined cellular environment and interact with intracellular targets, at the cost of a high predation risk to the attacking bacteria who seek close contact with a predator.

Coordinated attack behavior can mitigate some of this risk, flooding a predator with an overabundance of targets and overwhelming its prey-handling capacity to reduce individual predation risk, which is analogous to the scenario described in the selfish herd model (23). In addition, group coordination allows many bacteria to inject the predator simultaneously, pooling a large dose of virulent substances and rapidly shutting down predator attack capabilities.

Maintaining a sufficient attacker-to-predator ratio is an important factor in animal mobbing, allowing prey animals to achieve a local numeric superiority (7, 8). In bacteria, this function, as well as attack synchronization, may be carried out by quorum sensing signaling, which is used by bacteria to coordinate many communal efforts. Quorum sensing regulation is involved in the expression and secretion of enzymes and metabolites, biofilm formation and dispersal, expression of motility systems, and more (24). It works through the production and secretion of small signaling molecules. Extracellular signal concentrations correlating with bacterial population density are detected by specific receptors, activating downstream processes at specific signal thresholds. This regulatory architecture allows bacteria to invest into communal activities only when a sufficient quorum of potential collaborators is present and to synchronize these communal activities.

Bacteria are also able to sense chemical gradients and taxis toward or away from the source (i.e., chemotaxis [25]). In the context of predator attack behavior, sensing of a predator-specific secretion may enable bacteria to target a predator by taxing toward the source, building a high local population density of attackers. While P. aeruginosa peruses a similar strategy when invading Staphylococcus aureus microcolonies (26), this type of behavior was not reported previously in the context of predation.

The Pseudomonas quinolone signal (PQS) (27, 28) is a likely candidate for the regulation of predator attack behavior. This signal was shown to be involved in P. aeruginosa pathogenicity and stress response in a number of systems (29–31). This signal is unique to P. aeruginosa (32) and an important feature, allowing selective sensing to establish an attacking quorum of trustworthy (participating in attack behavior) and competent (express attack mechanisms) individuals.

Here, we look into the interaction of P. aeruginosa and Acanthamoeba castellanii, showing that the bacteria can sense amoebae from a distance and tax toward them, coordinate group attack behavior using the PQS signal, and produce an emergent behavior analogous in many ways to animal mobbing.

## RESULTS

### Time-lapse microscopy of Pseudomonas aeruginosa adhesion to and congregation on amoeba cells.

**FIG 1:**
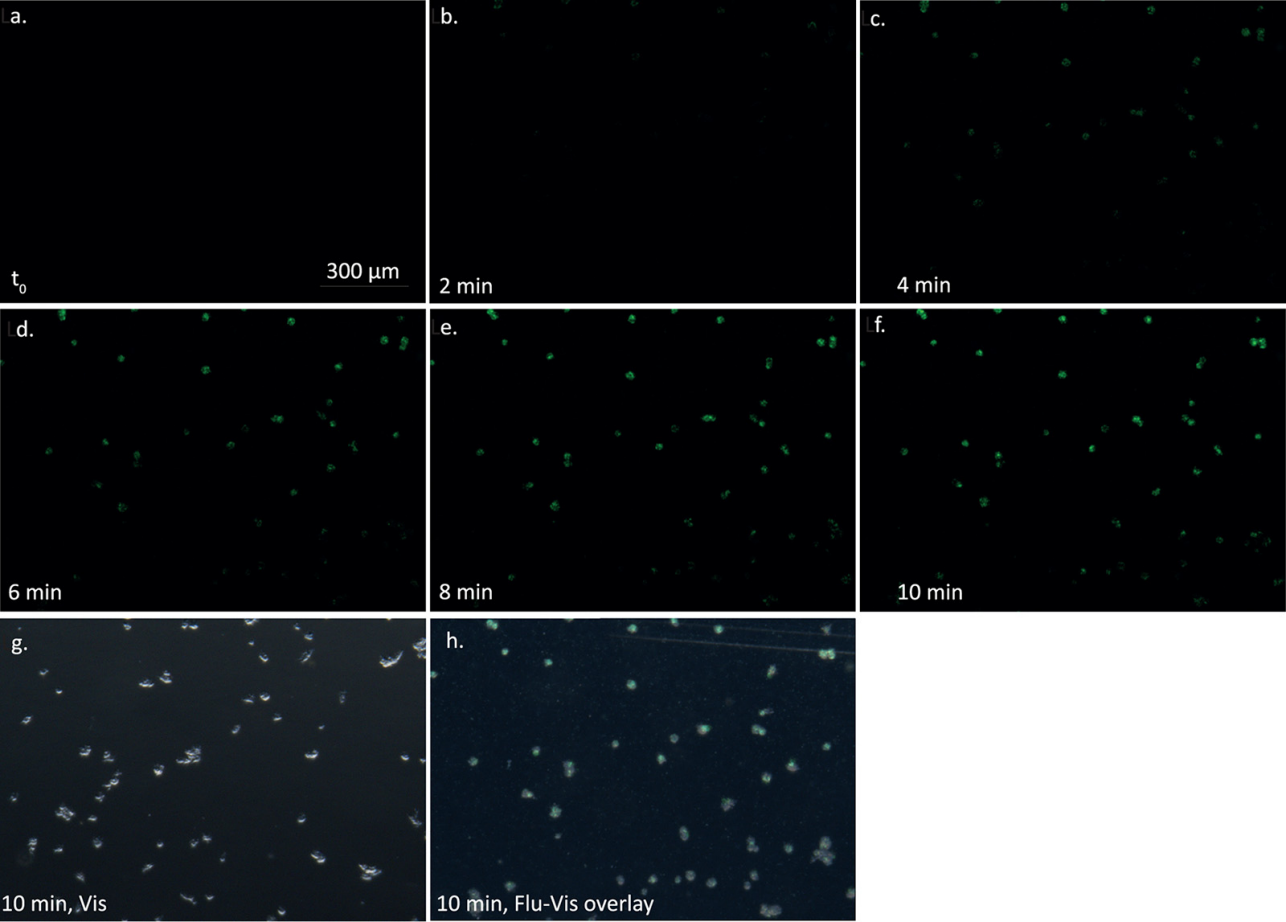
Time-lapse microscopy of adhesion of Pseudomonas aeruginosa to Acanthamoeba castellanii. (a to f) Time-lapse microscopy in green fluorescence protein (GFP) fluorescence wavelengths of bacterial adhesion to amoeba. (g) Bright-field image at 10-minute time point showing the position of amoeba cells within the field. (h) Superimposed image of 10-minute GFP fluorescence and bright-field to demonstrate the colocalization.

Time-lapse microscopy images are presented in Fig. 1. No fluorescence is seen at time 0 (t_0_), which is time at which the bacterial culture was injected into the cell (Fig. 1a). At 2 minutes, initial fluorescence can be observed as bacteria congregate on the amoeba cells (Fig. 1b). Fluorescence accumulates as more bacteria settle, reaching a plateau after 8 minutes (Fig. 1c to f). Superposition of a bright field image (Fig. 1g) and fluorescence at 10 minutes (Fig. 1f) is presented in Fig. 1h, showing colocalization of bacterial fluorescence and amoeba cells. Fluorescence is spread throughout the amoeba cells, focusing on the cells perimeters, and not in distinct locations within the cells, suggesting cell surface attachment and not phagocytosis. It is noteworthy that the signal is focused on the amoeba cells, suggesting a selective adhesion of the bacteria to the cell surface and not to the plastic surface of the counting chamber.

### Pseudomonas aeruginosa shows taxis toward amoebae.

**FIG 2:**
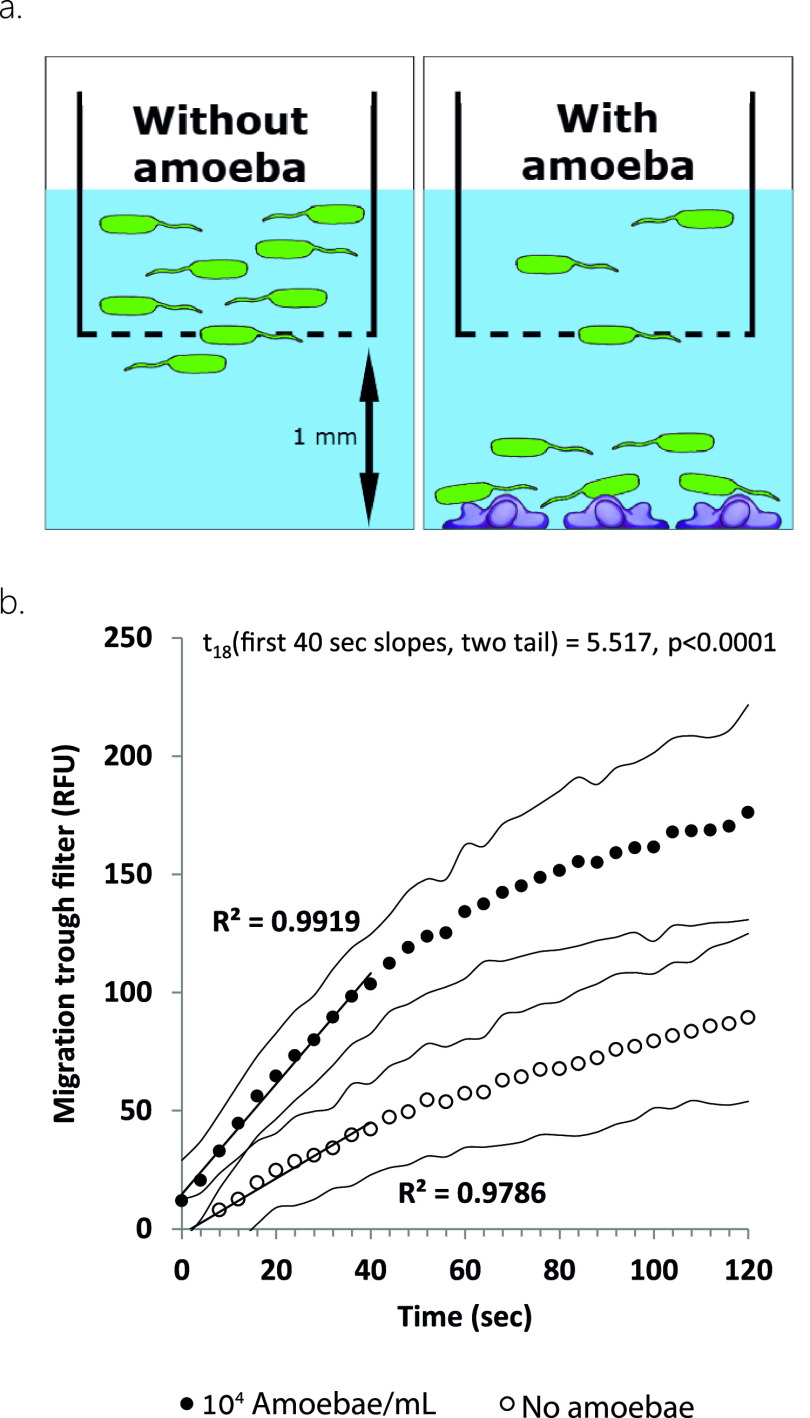
Sensation and attraction of Pseudomonas aeruginosa to amoebae. (a) Taxis measurement; the Fluoroblok system consists of a 24-well plate and a 3-μm pore size fluorescence blocking filter insert dividing each well into a top and a bottom chamber. Amoebae are added to the bottom chamber of a well and allowed to settle. GFP-tagged bacteria are added to the top chamber above the filter. Reading fluorescence from the bottom allows measurement of bacterial migration kinetics. (b) Migration kinetics of GFP-tagged PAO1 in the presence or absence of amoebae; *n* = 9 per treatment, circles stand for measurement times, flanking curves represent 1 SD.

To validate the ability of P. aeruginosa to sense amoebae and tax toward them, we used the Fluoroblok system, consisting of a 24-well plate and a fluorescence-blocking filter (Fig. 2a), allowing us to monitor kinetically the migration of green fluorescent protein (GFP)-tagged bacteria toward the amoebae. Migration of bacteria to the bottom chamber was faster when amoebae were present (Fig. 2a), starting at the first measurement of 4 seconds from the beginning of the experiment (Fig. 2b) (one-tailed *t* test, t_17_ = 2.18, *P* = 0.0215). This trend intensified as the experiment continued (Fig. 2b). Microscopy of samples taken after the kinetic measurement showed that amoebae did not separate from the well surface into the bulk liquid.

### Pseudomonas aeruginosa adhesion kinetics is dependent on amoeba population density.

**FIG 3:**
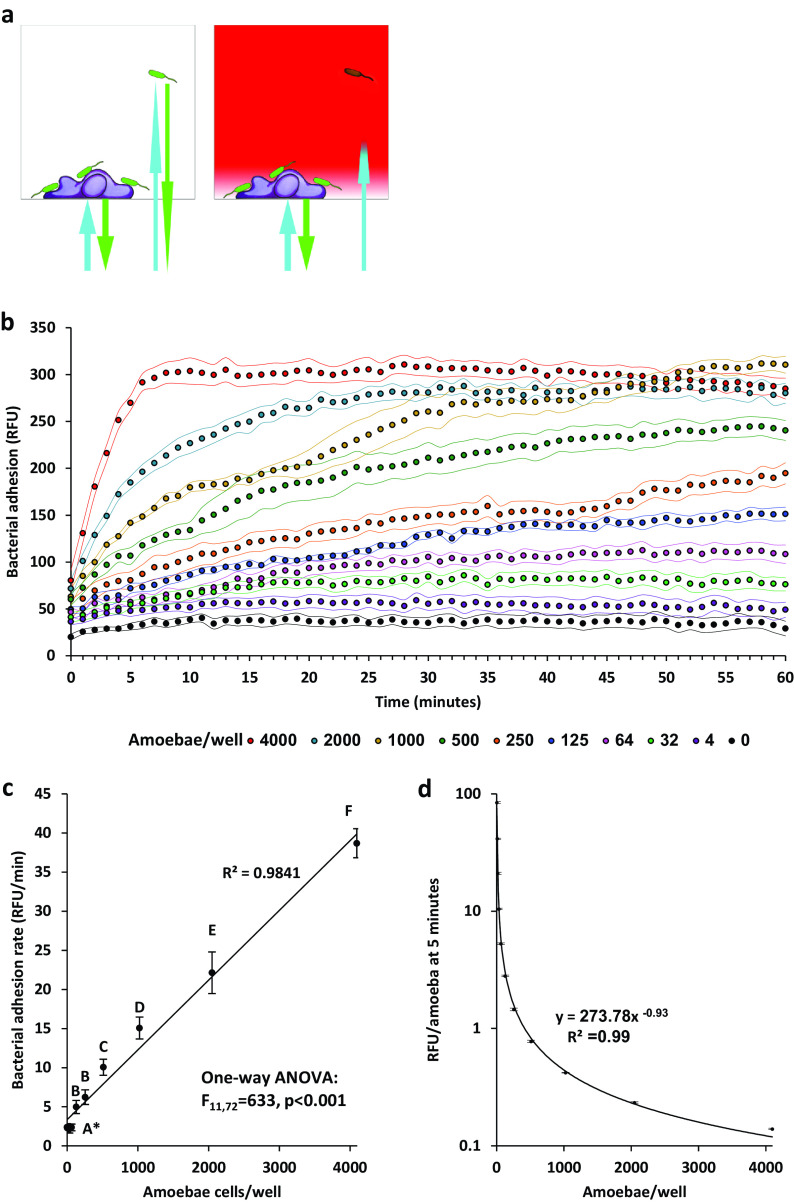
Bacterial adhesion kinetics at different amoeba population densities. (a) Conceptualization of adhesion kinetic measurement. In the absence of dye (left), light in GFP excitation and emission wavelengths travels freely through the microtiter well. The bottom fluorescence measurement under these conditions detects both attached and unattached bacteria. When the dye is added (right) light can penetrate only a few microns into the well, eliminating signal from bacteria in the bulk liquid, enabling a specific measurement of adhering bacteria (36). (b) Bacterial adhesion kinetics in different amoeba population densities; *n* = 7 per treatment, dots represent measurement, flanking curves represent ±1 SD. (c) Average adhesion rates at first 5 minutes. Different letters stand for significant differences at a *P* value of <0.05 according to an analysis of variance (ANOVA) test. (d) Adhesion rate per amoebae at first 5 minutes. *, Note that results for 0,4,8,16,32 and 64 amoeba per well (Panel c) were not significantly different (Tukey’s HSD post-hoc test).

The effect of amoeba population density on P. aeruginosa congregation was measured using a kinetic assay in a high-throughput setting (Fig. 3a) (33). Adhesion was found to be correlative to amoeba population density (Fig. 3b). Adhesion rates (relative fluorescence unit [RFU]/min) were in correlation with the number of amoeba (Fig. 3c). The number of bacteria that adhere to each amoeba (RFU/amoeba) was negatively correlated with the number of amoebae, following a power curve (Fig. 3d).

### Effect of nutrient clouding on adhesion to amoeba.

**FIG 4:**
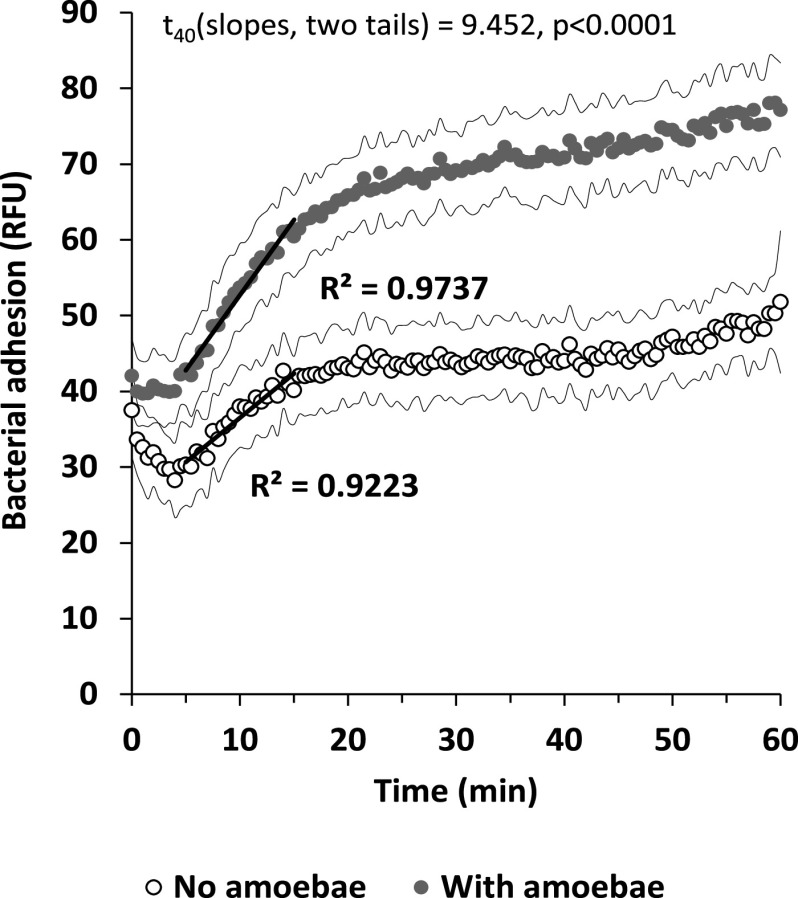
Effect of nutrient masking on Pseudomonas aeruginosa adhesion to amoebae. All wells contain LB Lennox medium, with or without 1,000 amoeba per well. *n* = 21 per treatment, error lines stand for ±1 SD.

The attraction of bacteria toward a bacterivore is counterintuitive. It may be part of a coordinated attack behavior, but it may also result from less specific and potentially fatal taxis toward nutrients in amoeba secretions. To eliminate the possibility that nutrients in the vicinity of the amoeba cell are driving bacterial taxis, we measured bacterial adhesion kinetics in the presence of soluble nutrients (LB medium). The abundance of nutrients should disrupt any nutrient gradient around the amoeba cells, disrupting directional movement. Results presented in Fig. 4 show that even in a nutrient-rich environment, amoeba induced faster and higher adhesion, suggesting that the observed attraction of P. aeruginosa to amoebae is driven by a predator-specific factor.

### Pseudomonas aeruginosa adhesion to amoebae is affected by PQS.

**FIG 5:**
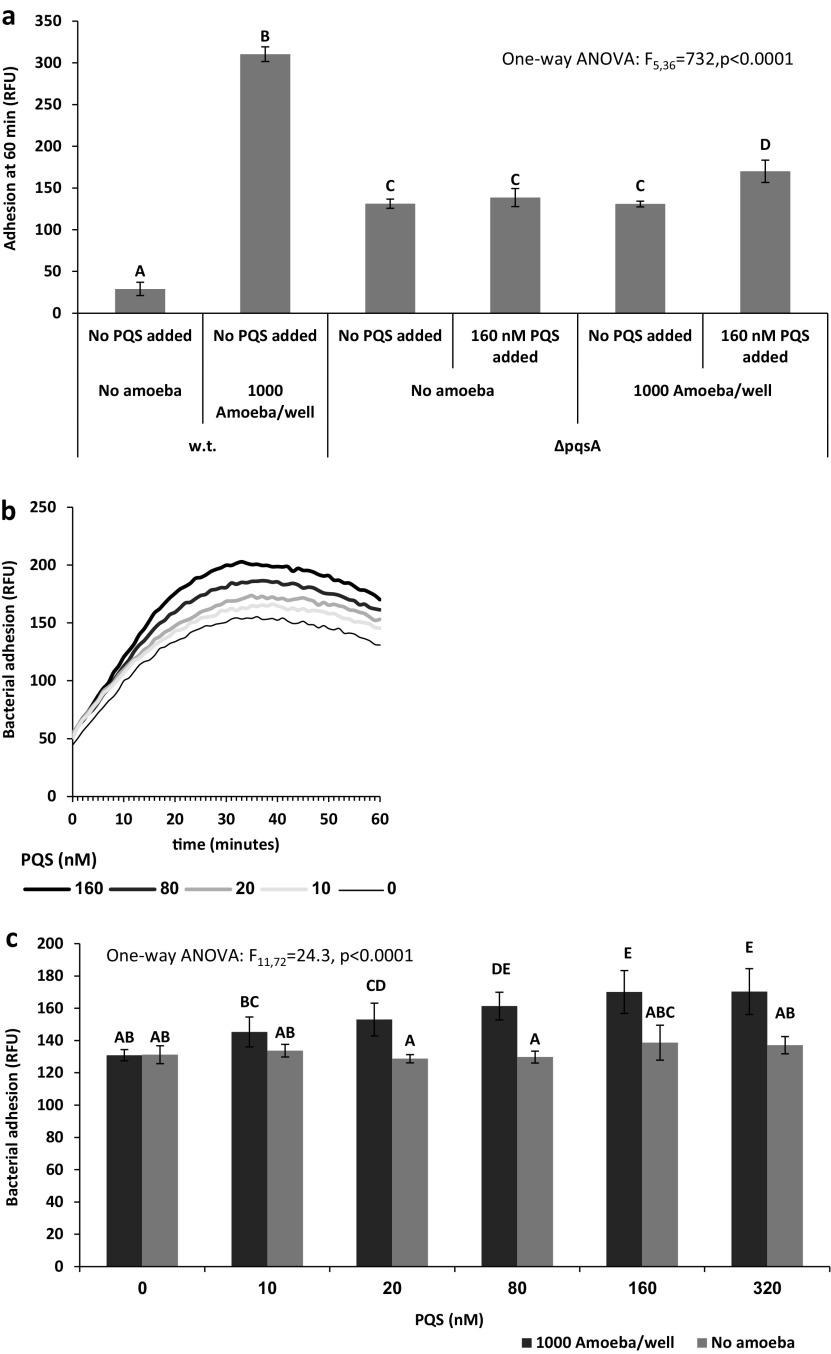
Effect of PQS on Pseudomonas aeruginosa adhesion to amoebae. (a) Adhesion kinetics of Δ*pqs*A in the presence of amoebae in different PQS concentrations. (b) WT and Δ*pqs*A adhesion at 60 minutes in the absence or presence of amoebae (1,000 per well), and with or without addition of 160 nM PQS. (c) Bacterial adhesion at 60 minutes in different PQS concentrations in the presence (dark gray) and the absence (light gray) of amoebae. In all cases, *n* = 7 per treatment and error bars stand for ±1 SD. Different letters denote statistically significant differences.

To test the effects of PQS on P. aeruginosa adhesion to amoebae, we used the Δ*pqs*A mutant, which is unable to produce 2-heptyl-4-quinolone (HHQ), a direct precursor of PQS. Adhesion behavior kinetics was measured in the presence and absence of amoebae and in different PQS concentrations. In the absence of amoebae wild type (WT), P. aeruginosa adhesion is low compared to the Δ*pqs*A mutant. Addition of 10,000 amoebae per mL induces a tenfold increase in w.t. adhesion, but has no effect on the mutant (Fig. 5a). Adding PQS increases Δ*pqs*A adhesion when amoebae are present but has no effect in their absence (Fig. 5a). It is noteworthy that the PQS effect is fast; 10 nM PQS produces a statistically significant effect within 1 minute (Fig. 5b) (one-tailed *t* test, t_13_ = 2.81, *P* = 0.007). The effect is dose dependent (Fig. 5b and c).

### PQS accumulation time scale.

**FIG 6:**
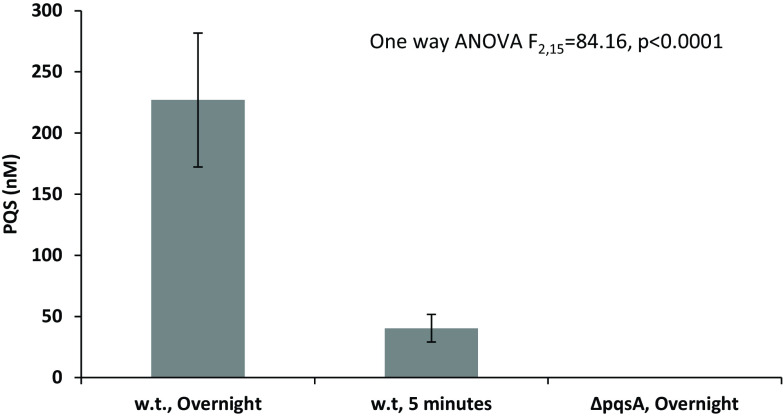
PQS signal accumulation in WT and Δ*pqs*A cultures. PAO1 WT and ΔpqsA overnight cultures were washed once in M9 buffer and resuspended and incubated in buffer for a period of 5 minutes. PQS was quantified in spent media and in buffer against a PQS standard using a bio-reporter (Pseudomonas putida expressing β-galactosidase under control *pqsR* promoter). *n* = 6 per treatment; error bars represent ±1 SD; all groups are statistically different from each other, *P* < 0.05.

The Δ*pqs*A mutant responded to externally supplied PQS. If this reaction is a natural part of P. aeruginosa behavior in the presence of amoebae, PQS should accumulate rapidly, allowing bacteria to estimate the number of currently available mobbing partners. Fig. 6 shows that an overnight WT culture diluted to an optical density at 600 nm (OD_600_) of 0.05 produces 40.4 ± 11.3 nM PQS within 5 minutes after the removal of all background signal. These levels are lower than overnight concentrations (227 ± 54.8 nM) but well within the range of concentrations inducing amoeba adhesion behavior (Fig. 5c).

### Survival of Pseudomonas aeruginosa WT and Δ*pqs*A under predation.

**FIG 7:**
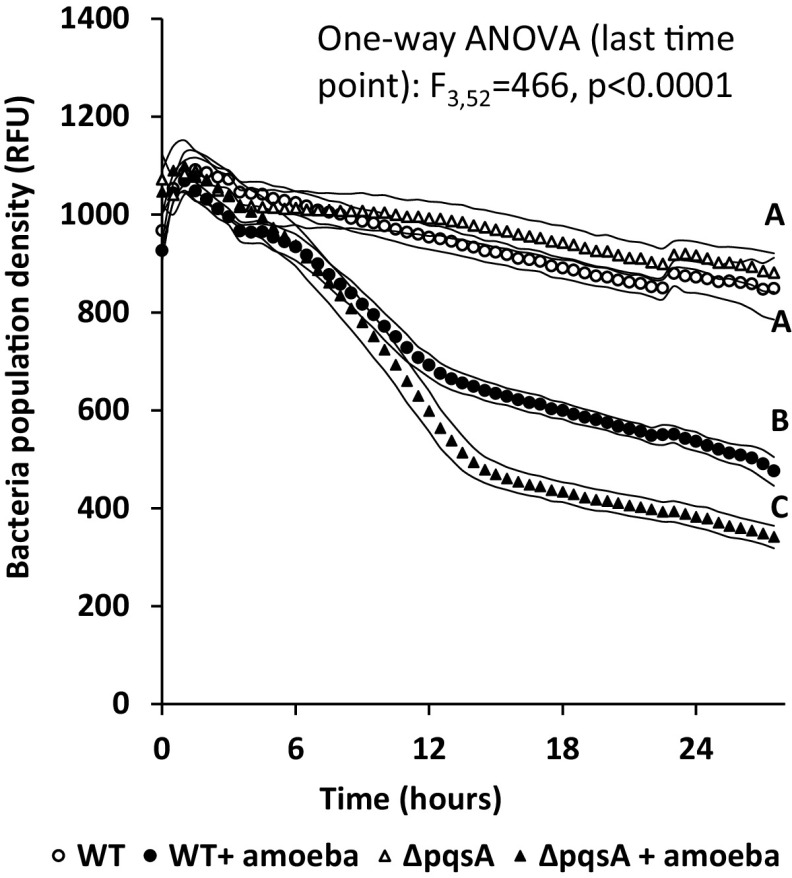
Population density kinetics of Pseudomonas aeruginosa WT (black) and Δ*pqs*A (gray) in the presence (closed symbols) and absence (open symbols) of amoebae; *n* = 14 per treatment, symbols signify measurement times, curves stand for ±1 SD. Different letters stand for statistically different groups at the last time point (*P* < 0.05).

PQS affects P. aeruginosa adhesion to amoeba, suggesting it has a role in bacterial predation avoidance. To test this suggestion, we followed bacterial population densities of P. aeruginosa WT and Δ*pqs*A in the presence and absence of amoebae over a period of 24 hours, using fluorescence as a bacterium-specific tag, as amoebae were found to affect OD (Fig. 7). No carbon source was added to the media in order to eliminate the effect of bacterial growth from the interaction. In the absence of amoebae, the population density of both the WT and mutant behaved similarly, and experienced a small decrease, probably due to starvation. In the presence of amoeba, the two strains diverged, with the Δ*pqs*A showing a more acute reduction in population. At the 24-h time point, the WT population was reduced by 44.01% ± 0.08%, while the mutant population was reduced by 61.31% ± 0.05%.

### Effect of bacterial prey on amoeba survival and growth.

**FIG 8:**
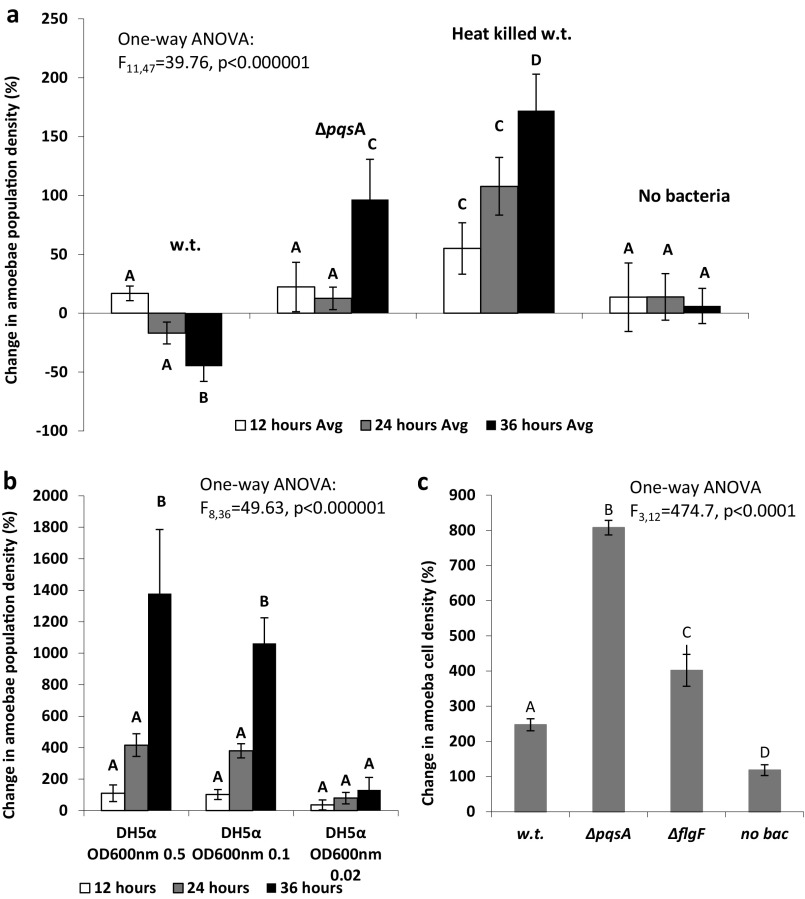
Effect of prey type on amoeba survival or growth. Amoebae were cocultured in microscopy counting chambers with various bacterial prey types. Bars show percent change in amoeba population density relative to t_0_; *n* = 5 per treatment, error bars stand for ± 1 SD. Different letters stand for significant differences at a *P* value of <0.05 according to an ANOVA test. (a) Pseudomonas aeruginosa WT and Δ*pqs*A; t_0_ amoeba population = 35 ± 5 (avg ± SD) per counting field; starting bacterial OD_600_ of 0.5 (1 cm) in all cases. (b*)* Escherichia coli DH5α; OD is calculated as OD_600_ (1 cm) after dilution into the counting chamber; t_0_ amoeba population = 35 ± 5 (avg. ± SD). (c) Pseudomonas aeruginosa WT, Δ*pqs*A, and Δ*flg*F—36-h time point; t_0_ amoeba population of 151 ± 19; starting bacterial OD_600_ = 0.5 (1 cm).

To test the effect of PQS signaling on the ability of P. aeruginosa to kill amoebae, we observed amoeba survival when coincubated with P. aeruginosa in disposable microscopy chambers. Wild-type PAO1 caused a reduction in amoeba population, while the heat-killed WT yielded an increase in amoeba population (Fig. 8a), showing that inhibition of amoeba growth is not the result of WT PAO1 having poor nutritional value. The Δ*pqs*A mutant yielded an intermediate result, supporting amoeba growth better than the WT but less than the heat-killed bacteria; PQS mediates the killing of amoebae. Escherichia coli DH5α, a prey species which does not attack the amoebae, supported a much greater amoeba growth (Fig. 8b), either due to it having a greater nutritional value or the absence of toxins contained in the heat-killed bacteria. To test whether motility is involved in the killing of amoebae, Δ*flg*F, a flagella-less strain, was also included in the experiment (Fig. 8c). This mutant shows an intermediate effect between the WT and the Δ*pqs*A, and swimming is important in restricting amoeba growth.

A comparison between the WT and the Δ*pqs*A in Fig. 8a and c demonstrates an interesting point that although both experiments show a similar trend, i.e., that the Δ*pqs*A mutant supports amoeba growth much better than the WT, the ratio of amoebae to bacteria is an important factor in the outcome. When the amoeba starting population was increased from 35 (Fig. 8a) to 150 cells (Fig. 8c), keeping the bacterial population constant, amoeba success was much greater.

## DISCUSSION

Pseudomonas aeruginosa engages in active predator attack behavior, coordinated by the PQS quorum sensing signal, facilitating predation avoidance. The system is more complex than a simple predator-prey interaction—P. aeruginosa is able to kill the amoebae and possibly feed on them. The outcome of the interaction is dependent on the population ratio of predator to prey, suggesting that neither side holds an overwhelming advantage over the other nor is able to out compete it in all scenarios. The Pseudomonas quinolone signal played a part in the ability of bacteria to hold amoeba growth, possibly by coordination of bacterial swimming and adhesion behavior. Killing of amoebae by P. aeruginosa was shown previously to involve the type 3 secretion system (20, 21), requiring swimming and adhering to the predator (34), putting attacking bacteria in high predation risk. Numeric superiority and synchronization of attack are adaptations employed by many animal species to overcome predator superiority (14) and may also be applicable in this case. Indeed, time-lapse microscopy and adhesion experiments with the WT and QS mutant showed that bacterial adhesion is fast (minute time scale), specific (high preference to the amoebae and not to the plastic surface), and PQS dependent. Migration experiments have shown positive taxis of the bacteria toward the amoebae, demonstrating that bacteria are able to sense and are attracted to amoebae from a distance. Flagellum-less bacteria showed reduced survival under predation, supporting the utility of swimming in predation avoidance. The attraction of the bacteria to the amoebae was not suppressed by the addition of nutrients, suggesting that bacteria respond to a predator-specific signal and not to a nutrient-rich environment around the amoebae. Maximum adhered bacterial fluorescence (i.e., measured with dye) was only one-third of the total fluorescence (measured without dye), suggesting the attack was carried out by a subpopulation as described previously (35).

Bacterial group behavior is often coordinated by QS signals, small molecules secreted and sensed by bacteria (24). Pseudomonas aeruginosa expresses three of these systems, namely, the Las, Rhl, and PQS. The Las and Rhl QS systems are based on *N*-acyl homoserine lactones, which were found to facilitate QS in many bacterial species (24) and allow interspecies cross talk (36), making these two systems unsuitable in the context of bacterial predator attack where the signal has to be phylogenetic specific to detect only individuals able to participate in the attack. Indeed, the Las and Rhl signals were tested and found not to be involved in A. castellanii attack, at least on the hour time scale (22). The PQS system is P. aeruginosa specific (32), making it a very suitable signal for the presence of capable mobbing partners. Indeed, here, we show that predator adhesion behavior and attack success are PQS dependent, with the Δ*pqs*A mutant strain showing no amoeba-induced adhesion and a lesser amoeba-killing ability than the wild type. Adding PQS signal to the mutant restores some of the mutant adhesion behavior in a dose-dependent manner, but only in the presence of amoebae, further supporting the idea that bacteria modify their predator attack behavior based on the perceived number of conspecifics and predators. The effect of externally added PQS is very fast, within 1 minute of adding the signal, fitting well with the requirement for a fast response and with the very fast accumulation of PQS signal in washed WT cells. These response times suggest that PQS activity in this case is independent of protein expression, the canonical QS mode of action. Other works have shown that a considerable portion of PQS-responding genes are independent of the *pqsR* gene (37), which is the only known receptor for this signal. Our results show that PQS signaling regulates fast behavioral responses, suggesting the existence of a pathway that works independently of gene expression.

In summary, here, we show bacterial mobbing-like behavior, where a group of bacteria enact a coordinated attack behavior toward a predator, similar to mobbing behavior seen in animals, responding to predator population density (as manifested by PQS signal) and predator secretions, producing a fast response. This behavior, based in predation avoidance, may be the setting from which many QS-dependent pathogenicity mechanisms emerge, evolved to deal with protozoan predators, but applicable to phagocytic components of immune systems of multicellular organisms.

## MATERIALS AND METHODS

### Bacterial strains and plasmids.

Pseudomonas aeruginosa PAO1 WT and P. aeruginosa PAO1 Δ*pqs*A (38), carrying the pMRP9-1 plasmid encoding carbenicillin resistance and constitutive expression of GFP_mut2_ (39, 40), were cultivated in 50 mL of M9 medium (47.75 mM Na_2_HPO_4_, 22.05 mM KH_2_PO_4_, 8.56 mM NaCl, 18.69 mM NH_4_Cl, 2 mM MgSO_4_, and 0.1 mM CaCl_2_), supplemented with 200 μg/mL carbenicillin and 0.4% glucose in 100-mL Erlenmeyer flasks, at 37°C with 120 rpm for a period of 16 hours. Before each experiment, cultures were centrifuged (5,000 × *g* for 1 minute), washed once with Tris-buffered salt solution (TBSS; 2 mM KCl, 1 mM CaCl_2_, 0.5 mM MgCl_2_, and 1 mM Tris), and resuspended in the TBSS to the OD_600_ specified in the experiment description below.

### Amoeba culture conditions.

Acanthamoeba castellanii was cultured in 10 mL of peptone-yeast-glucose (PYG) medium (ATCC 712) supplemented with 100 μg/mL gentamicin, in 50-mL tissue culture flasks (Greiner, Germany) at 25°C, without shaking, for 5 days. To harvest the cells, a flask was shaken vigorously to separate the amoebae and 9 mL was transferred to 1.5-mL plastic microtubes (1.5 mL in each tube). The tubes were centrifuged (200 × *g* for 30 sec) to collect the cells. The liquid was replaced gradually by TBSS; 500 μL was replaced with TBSS at the first stage, incubated for 5 minutes, separated once more by replacing 1,000 μL at the second stage, incubated for an additional 5 minutes, and then separating and replacing the full content of the tube (1,500 μL). Following a full replacement of PYG with TBSS, the tubes were centrifuged once more and cell pellets collected into 1 mL of TBSS in a 10-mm glass tube. Cell density was determined by microscopy in a disposable penta-square counting chamber (Vacutest Kima, Italy; used throughout this work) and diluted in TBSS to the cell densities specified in the experiment description below.

### Time-lapse microscopy of Pseudomonas aeruginosa adhesion to Acanthamoeba castellanii.

A total of 10 μl of amoeba at a culture density of 5 × 10^5^ cells/mL in TBSS medium was added to a counting chamber and placed under an Epifluorescence binocular system (Nikon SMZ18 connected to a Nikon DS-Fi3 camera). Cells were imaged in visible light and in green fluorescence at ×12 magnification, 500-ms exposure time, gain of 14×, field size of 2,880 by 2,048 pixels, and dynamic range of 3 by 8 bit. These setting were chosen to acquire only aggregated GFP-expressing bacteria but exclude the signal produced by planktonic bacteria. At the start of each experiment, 10 μL of the GFP-expressing WT P. aeruginosa PAO1 culture was added. An image was acquired in green fluorescence every 10 seconds for a period on 10 minutes. At the 10-minutes time point, bright field images were also captured to validate the location of the amoebae and colocalization with bacterial fluorescence.

### Pseudomonas aeruginosa taxis toward amoebae.

Taxis was tested using the Corning FluoroBlokHTS 24-well multiwell permeable support system with a 3.0-μm high density polyester (PET) membrane (Corning, New York, USA) as described in Fig. 2. This filter system was chosen to allow the transfer of bacteria but not of amoeba. The plates were measured using a Synergy HT plate reader (Biotek, USA; used throughout this work).

Pseudomonas aeruginosa and amoeba were cultivated and prepared as described above. Bacteria were diluted to OD_600_ of 0.1, and amoebae were diluted to 1 × 10^4^ cells/mL (both in TBSS). A total of 750 μL of the amoeba suspension was added to the bottom chambers of columns 1 to 3 of the base plate, columns 4 to 6 were loaded with 750 μl of TBSS buffer and used as the control, and the filter system was mounted onto the base plate. The top chamber of a single well was loaded with 100 μl of the bacterial culture and read kinetically for bottom fluorescence every 4 seconds for 2 minutes, using the following reading parameters: excitation, 485 nm/20; emission, 528 nm/20; and gain, 60. The process was repeated for all wells. At the end of the experiment, the filter insert was removed and five wells were sampled (10 μL each from the bulk liquid away from the bottom of the well) and examined microscopically to verify that amoebae did not detach from the bottom of the well.

### Pseudomonas aeruginosa short-term adhesion kinetics in various amoeba cell densities.

Bacterial adhesion to amoebae was quantified using a kinetic assay in a 96-well plate (33) (Fig. 3). A total of 50 μL of an amoeba suspension (8 × 10^4^ cells/mL TBSS) was loaded into the first column of a 96-well plate (clear polystyrene, flat bottom; Jet-biofil, China), diluted in a double dilution series using TBSS as described in Table 1, and left to settle on the plate for 1 hour.

**TABLE 1:**
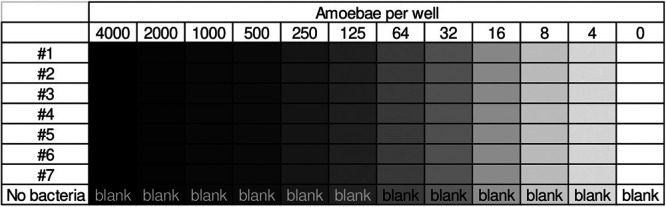


Pseudomonas aeruginosa PAO1 WT was cultured, washed, resuspended in TBSS, diluted to an OD_600_ of 0.1 (as determined for 100 μl in a 96-well plate, equivalent to 0.33 for 1 cm), and supplemented with 1.6 mg/mL Red#40 (Sigma, Israel). A total of 50 μL of this culture was pipetted into rows A to G of the plate containing the amoeba. Row H was added with TBSS, supplemented with 1.6 mg/ml dye (no bacteria). These wells were used as blanks. Addition of the bacterial culture was performed within 30 seconds, using a multichannel pipettor, to capture adhesion from its very beginning. The final bacterial culture density in the plate was an OD_600_ of 0.05 and the dye concentration was 0.8 mg/mL. The plate was loaded into the plate reader and read kinetically for bottom fluorescence in 1-minute intervals for 60 minutes (excitation, 485/20; emission, 528/20; gain, 60).

### Testing the effect of nutrients on adhesion to amoeba.

Pseudomonas aeruginosa was grown as above but in LB medium to avoid the effects of a dramatic change in media. The bacteria were washed and suspended in fresh LB with 1.6-mg/ml Allura red to an OD_600_ of 0.1. Amoeba were washed and suspended in LB to 20,000 per ml, and 50 μl was placed in a 96-well plate in columns 1 to 3, giving 1,000 amoeba cells per well. Columns 4 to 6 were filled with 50 μl of sterile LB. To wells 1 to 6 of row H, 50 μL of LB supplemented with 1.6 mg/mL Allura red was added to form blanks. A total of 50 μl of a bacterium-dye suspension was added to rows A to G of columns 1 to 6 using a multichannel pipettor, and bacterial adhesion kinetics was read as described earlier.

### Effect of the Pseudomonas Quinolone Signal (PQS) on Pseudomonas aeruginosa amoeba adhesion.

Pseudomonas aeruginosa PQS (2-nonyl-3-hydroxy-4-quinolone; Sigma, Israel) was dissolved in dimethyl sulfoxide (DMSO) to a concentration of 10 mM and diluted in TBSS medium in a double dilution series to resulting concentrations of 20 μM to 20 nM per well (11 concentrations + negative control) in a volume of 25 μL. An amoeba suspension in TBSS (4 × 10^4^ cells/mL) was added to each well (25 μL per well), to result in 1,000 cells per well. Pseudomonas aeruginosa PAO1 Δ*pqs*A-expressing GFP_mut2_ was cultivated and prepared as described above and diluted to an OD_600_ at 0.1 in TBSS supplemented with 1.6 mg/mL Red#40. From this suspension, 50 μL was added to rows A to G of the amoeba-PQS plate, to a final bacterial density of OD_600_ of 0.05, dye concentration of 0.8 mg/mL, 1,000 amoebae per well, and 5 μM to 5 nM PQS. Row H was loaded with dye supplemented TBSS and used as blanks. The plate was loaded on the plate reader and read for bottom fluorescence every minute for a period of 1 hour. The experiment was repeated without amoebae using the same PQS concentrations (amoebae were replaced with an additional 25 μL of TBSS per well).

### Predation avoidance of Pseudomonas aeruginosa WT and Δ*pqs*A.

Amoebae were suspended and diluted in TBSS to 2 × 10^4^ cells/mL. A total of 50 μL of the above suspension (1,000 amoeba) was pipetted into columns 1 to 4 a flat-bottomed, clear 96-well plate. Columns 5 to 8 were loaded with sterile TBSS. Pseudomonas aeruginosa PAO1 WT and Δ*pqs*A mutant, both expressing GFP_mut2_, were cultured and washed as described above and diluted to an OD_600_ of 0.1. The wild type was added to wells A to G of columns 1 to 2 and 5 to 6, and the Δ*pqs*A was added to wells A to G of columns 3 to 4 and 7 to 8 (*n* = 14 per treatment group). Bacterial predation was estimated by the disappearance of GFP fluorescence; a bottom fluorescence reading was taken every 30 minutes over a period of 24 hours.

### Effect of bacterial strain and population density on amoeba population.

PAO1 WT, Δ*pqs*A, and Δ*flg*F (41) were cultured overnight in M9 medium. Escherichia coli DH5α was cultured in Lennox LB (Himedia, Mumbai, India). All strains were washed twice in M9 buffer and diluted in M9 buffer to 10-fold the final designated concertation in TBSS buffer. A portion of the PAO1 WT culture was washed once in M9 buffer, transferred to 1.5-mL plastic microtubes, and heat killed at 65°C for 20 minutes. A sample of the heat-killed bacteria was plated onto an LB plate to verify inactivation. Acanthamoeba castellanii was cultivated, washed, measured as described previously, and then diluted to 2 × 10^5^ cells per mL in TBSS. A total of 27 μl of the amoeba suspension was pipetted into all cells of six plastic counting chambers. Chambers were added with 3 μL of either the live or heat-killed bacterial suspension in TBSS buffer to give the designated final OD_600_ or in TBSS only (no bacterial control). Each condition was tested in 5 replicates. Counting chambers were kept in a humidified chamber at 25°C, and amoebae were counted microscopically at the designated times. Results are given as average % change in the number of amoebae from t_0_.

### Temporal quantification of PQS secretion.

The release of PQS from PAO1 WT and Δ*pqs*A (as negative control) was followed in order to see if relevant PQS concentrations build in a short time frame to support the regulation of fast behaviors. To this end, the bacteria were grown overnight in M9 media and the OD_600_ was determined; bacteria were separated from medium by centrifugation as above and spent media were collected for analysis. Cells were washed once and suspended in M9 buffer, incubated for 5 minutes in 25°C, and removed from the medium which was collected for analysis. Pseudomonas quinolone signal accumulation in an overnight culture media was quantified using a bio-reporters strain based on Pseudomonas putida, where PQS presence is manifested as β-galactosidase activity in a dose-dependent manner, adapted from Müller and Fetzner 2013 (42). Pseudomonas quinolone signal was also measured five minutes after bacteria were washed and transfered into fresh buffer in order to quantify PQS accumulation over short periods of time. Briefly, reporter cells were cultured overnight (28°C and 200 rpm) in LB supplemented with 50 μg/mL kanamycin, diluted in LB medium (2×), and supplemented with 100 μg/mL kanamycin to an OD_600_ of 0.1. Spent medium/buffer samples were serially diluted in order to bring them within the linear range of the reporter assay. The standard was prepared from a 10 mM PQS solution (Cayman Chemicals, MI, USA) in methanol and diluted to 80, 40, 20, 10, and 5 nM in M9 buffer. Samples and standards were mixed in a 1:1 ratio with the bio-reporter culture (total volume of 2 mL) and incubated for 6 hours (28°C and 200 rpm). A total of 20 μL from each sample was added to a 10-mm glass tubes containing 800 μL of Z buffer (60 mM Na_2_HPO_4_, 40 mM NaH_2_PO_4_, 10 mM KCl, 1 mM MgSO_4_, and 50 mM 2-mercaptoethanol), 20 μL 0.1% (vol/vol) SDS, and 20 μL chloroform. They were incubated for a period of 15 minutes (28°C and 200 rpm) to release β-galactosidase. A total of 200 μL of each sample was transferred to a 96-well plate (clear polystyrene, flat bottom, tissue culture [TC] treated; SPS, South Korea). A total of 50 μL of 4 mg/mL ortho-nitrophenol-beta-d-galactopyranoside (ONPG) (Sigma, Israel) in Z buffer was added to each well. The plate was loaded onto the plate reader and read kinetically for the OD_420_ value every minute for half an hour. Slopes were calculated for each sample as well as for the standard to extrapolate PQ concentrations in samples.

### Statistical analysis.

The statistical analysis was conducted using the astatsa.com calculator (43). Where present, the *post hoc* analysis used Tukey’s honestly significant difference (HSD) test.
